# Supplementary material for: MAPK4 promotes triple negative breast cancer growth and reduces tumor sensitivity to PI3K blockade
Source: Nat Commun. 2022 Jan 11;13:245. doi: 10.1038/s41467-021-27921-1 (PMC8752662; doi:10.1038/s41467-021-27921-1)
Supplement: Supplementary file 1 — Supplementary Information [file 41467_2021_27921_MOESM1_ESM.pdf]

Supplementary Information

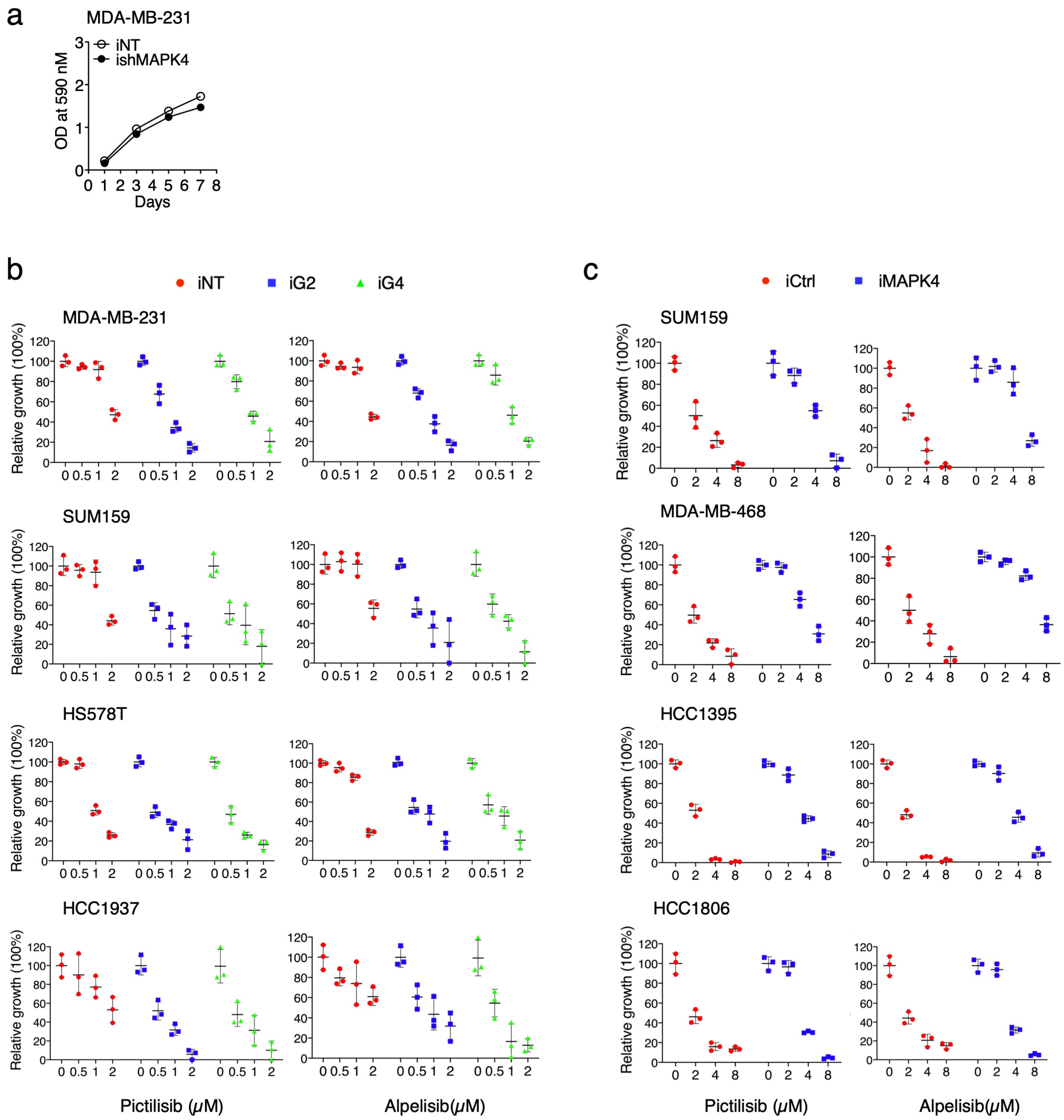

**Supplementary Fig. S1 MAPK4 regulation of TNBC cells. a** Proliferation assays comparing the growth of engineered MDA-MB-231 cells with 4 μg/ml Dox-induced knockdown of MAPK4 (ishMAPK4) or control (iNT). **b** The relative growth of MAPK4-knockdown TNBC cells as described in Fig. 7a-d. **c** The relative growth of MAPK4-overexpressing TNBC cells as described in Fig. 8a-d. All vehicle treated arms were set at 100% to provide relative growth inhibition by PI3K inhibitors in these differentially engineered TNBC cells.

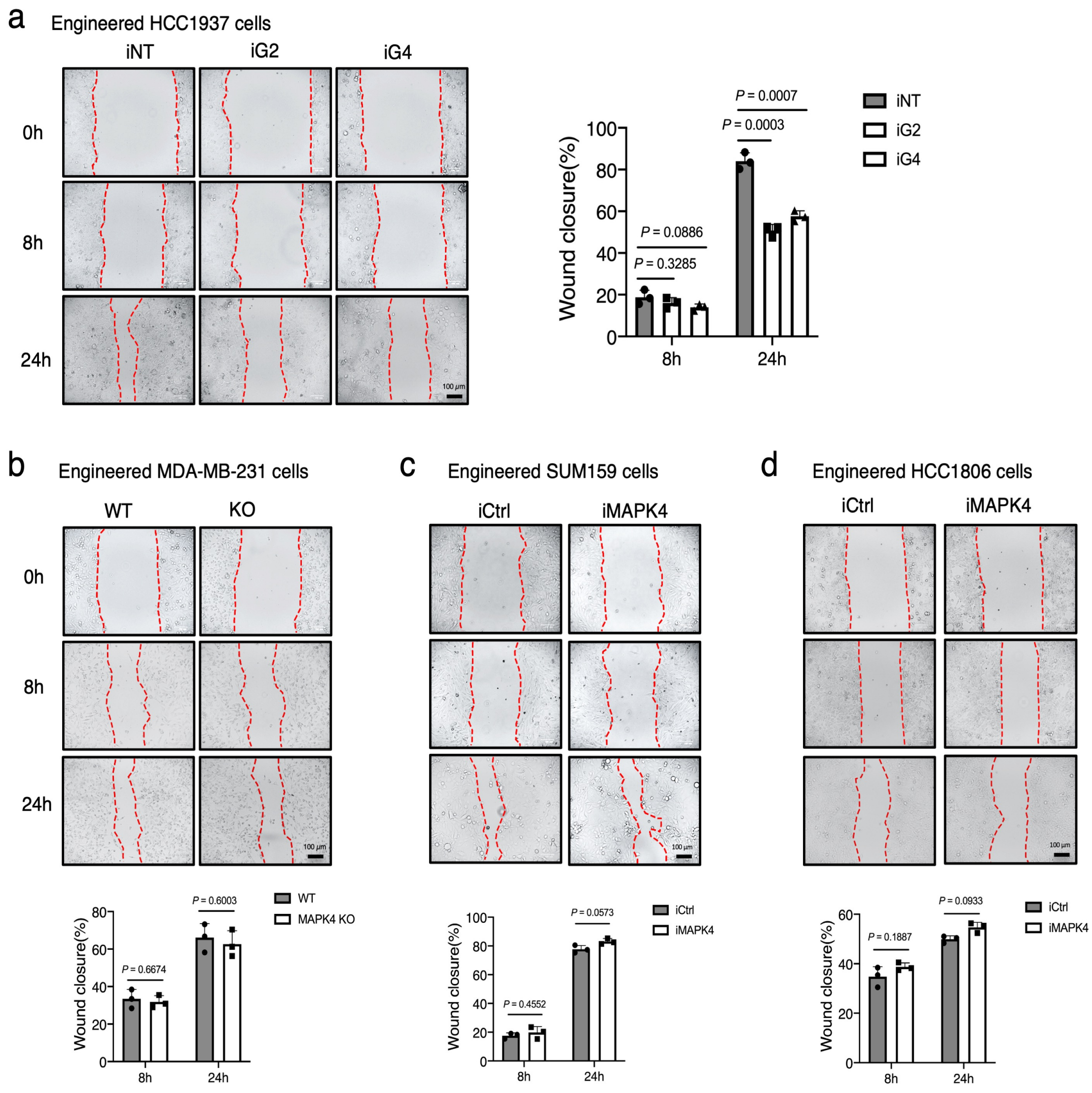


**Supplementary Fig. S2 MAPK4 regulation of TNBC cell migration.** Wound healing assays on the engineered **a** H1937 cells with 4 μg/ml Dox induced knockdown of MAPK4 (iG2, iG4) or control (iNT), **b** wild type (WT) and *MAPK4*-knockout (KO, clone #3) MDA-MB-231 cells, and **c,d** SUM159 and HCC1806 cells with 0.5 μg/ml Dox induced overexpression of MAPK4 (iMAPK4) or control (iCtrl). Representative images of cells after 0, 8h, and 24h after the initial scratch were shown. Bar: 100 μm. Also shown are the quantification (mean ± SD) of percentage of wound closure at 8h and 24h after scratch. *P* values determined by unpaired 2-tailed Student's *t* test. Data are representative of at least 2-3 independent experiments.

Source Data for supplementary Fig. 1a

| Day | iNT | | | ishMAPK4 (G4) | | |
|---|---|---|---|---|---|---|
| 1 | 0.226 | 0.211 | 0.21 | 0.172 | 0.163 | 0.14 |
| 3 | 0.925 | 0.99 | 0.973 | 0.897 | 0.851 | 0.773 |
| 5 | 1.42 | 1.366 | 1.358 | 1.301 | 1.196 | 1.23 |
| 7 | 1.762 | 1.693 | 1.721 | 1.417 | 1.469 | 1.522 |

Source Data for supplementary Fig. 1b

MDA-MB-231

| Pictilisib | iNT | | | iG2 | | | iG4 | | |
|---|---|---|---|---|---|---|---|---|---|
| 0 | 105.7 | 99 | 95.38 | 104.5 | 96.3 | 99.18 | 107 | 97.19 | 96.3 |
| 0.5 | 93.92 | 92.4 | 96.94 | 68.74 | 76.3 | 58.04 | 84.2 | 72.01 | 83.4 |
| 1 | 93.97 | 82.8 | 98.61 | 39.49 | 30.8 | 33.27 | 48.4 | 48.88 | 40.1 |
| 2 | 47.54 | 42.1 | 52.18 | 10.33 | 14.1 | 18.83 | 12.1 | 32.93 | 17.2 |
| Alpelisib | iNT | | | iG2 | | | iG4 | | |
| 0 | 105.7 | 99 | 95.38 | 104.5 | 96.3 | 99.18 | 107 | 97.19 | 96.3 |
| 0.5 | 92.86 | 92.7 | 98.05 | 63.25 | 68.7 | 72.3 | 81.5 | 96.96 | 79 |
| 1 | 92.2 | 101 | 87.77 | 44.88 | 29.7 | 38.21 | 38.6 | 44.66 | 55 |
| 2 | 42.09 | 44 | 47.49 | 20.66 | 17.7 | 10.88 | 23 | 16.07 | 22.1 |

SUM159

| Pictilisib | iNT | | | iG2 | | | iG4 | | |
|---|---|---|---|---|---|---|---|---|---|
| 0 | 111 | 92.5 | 96.51 | 104.5 | 96.9 | 98.62 | 95.3 | 113.6 | 91.1 |
| 0.5 | 96.51 | 101 | 89.68 | 45.58 | 57.4 | 60.71 | 64.3 | 46.2 | 43.6 |
| 1 | 104 | 97.2 | 79.64 | 50.88 | 19.3 | 37.52 | 23.4 | 61.68 | 33.4 |
| 2 | 48.95 | 40.3 | 43.1 | 18.07 | 27.3 | 39.88 | 20.6 | 33.6 | 0 |

| Alpelisib | iNT | | | iG2 | | | iG4 | | |
|---|---|---|---|---|---|---|---|---|---|
| 0 | 111 | 92.5 | 96.51 | 104.5 | 96.9 | 98.62 | 95.3 | 113.6 | 91.1 |
| 0.5 | 93.03 | 112 | 103.6 | 65.03 | 50.9 | 48.53 | 68.5 | 62.34 | 48.4 |
| 1 | 87.73 | 103 | 110.3 | 50.88 | 17.9 | 37.52 | 44.4 | 34.93 | 48 |
| 2 | 59.55 | 61.2 | 45.75 | 0 | 18.5 | 44.2 | 0 | 11.94 | 22.3 |

HS578T

| Pictilisib | iNT | | | iG2 | | | iG4 | | |
|---|---|---|---|---|---|---|---|---|---|
| 0 | 102.8 | 99.6 | 97.52 | 105.2 | 95.4 | 99.38 | 105 | 95.24 | 100 |
| 0.5 | 97.22 | 103 | 93.85 | 44.74 | 47.7 | 54.81 | 47.4 | 55.19 | 38.4 |
| 1 | 47.15 | 56 | 49.42 | 32.15 | 40.4 | 37.94 | 29.2 | 25.1 | 23.8 |
| 2 | 28.8 | 23.7 | 25.19 | 11.25 | 22.3 | 30.3 | 19 | 18.91 | 10.8 |

| Alpelisib | | iNT | | iG2 | | | iG4 | | |
|---|---|---|---|---|---|---|---|---|---|
| 0 | 102.8 | 99.6 | 97.52 | 105.2 | 97.7 | 99.38 | 105 | 95.24 | 100 |
| 0.5 | 100.2 | 94.5 | 91.57 | 62.53 | 49.7 | 51.12 | 50.8 | 52.35 | 68.2 |
| 1 | 81.92 | 88 | 86.41 | 54.81 | 49.6 | 38.44 | 34.5 | 50.63 | 51.7 |
| 2 | 31.4 | 25.5 | 29.34 | 18.55 | 27.9 | 12.67 | 19.7 | 30.26 | 12.5 |

HCC1937

| Pictilisib | | iNT | | iG2 | | | iG4 | | |
|---|---|---|---|---|---|---|---|---|---|
| 0 | 87.51 | 101 | 111.7 | 111.5 | 95.4 | 93.16 | 90.4 | 121.9 | 87.7 |
| 0.5 | 69.75 | 87.7 | 112.9 | 63.47 | 48.3 | 44.53 | 41.3 | 62.57 | 40.1 |
| 1 | 66.2 | 89.1 | 76.37 | 26.84 | 38.2 | 30 | 30.2 | 47.6 | 15.9 |
| 2 | 39.24 | 53.6 | 66.52 | 7.263 | 0 | 10.11 | 0 | 11.08 | 19.2 |
| Alpelisib | | iNT | | iG2 | | | iG4 | | |
| 0 | 87.51 | 101 | 112.2 | 111.5 | 95.4 | 93.16 | 90.4 | 120.7 | 87.7 |
| 0.5 | 73.79 | 76.4 | 88.64 | 48.63 | 72.6 | 60.32 | 58.1 | 39.52 | 66.2 |
| 1 | 53.12 | 73.1 | 95.59 | 31.89 | 61.3 | 37.58 | 12.6 | 35.63 | 1.5 |
| 2 | 54.57 | 57.5 | 70.88 | 33.79 | 44.8 | 16.74 | 20.1 | 7.186 | 11.4 |

Source Data for supplementary Fig. 1c

SUM159

| Pictilisib | | iCtrl | | iMAPK4 | | |
|---|---|---|---|---|---|---|
| 0 | 101 | 93.2 | 105.9 | 101.8 | 87.8 | 110 |
| 0.5 | 39 | 63.8 | 47.68 | 92.3 | 80.1 | 92.5 |
| 1 | 33.5 | 24.8 | 20.87 | 55.14 | 49.3 | 60.2 |
| 2 | 3.91 | 5.22 | 0.435 | 8.569 | 12.8 | 0.28 |
| Alpelisib | | iCtrl | | iMAPK4 | | |
| 0 | 101 | 93.2 | 105.9 | 101.8 | 87.8 | 110 |
| 0.5 | 53.5 | 62.5 | 48.99 | 107.9 | 96.5 | 101 |
| 1 | 4.93 | 17.1 | 28.55 | 74.05 | 83.1 | 100 |
| 2 | 0 | 0 | 4.058 | 21.98 | 33.1 | 25.8 |

MDA-MB-468

| Pictilisib | | iCtrl | | iMAPK4 | | |
|---|---|---|---|---|---|---|
| 0 | 98.4 | 93 | 108.6 | 99.98 | 104 | 95.5 |
| 2 | 47.1 | 58.7 | 43.24 | 101.8 | 92.4 | 98.5 |
| 4 | 24.4 | 24.2 | 16.87 | 65.64 | 72 | 58.6 |
| 8 | 10.1 | 14.9 | 0.331 | 38.63 | 24 | 30 |

| Alpelisib | | | iCtrl | | | iMAPK4 | |
|---|---|---|---|---|---|---|---|
| | 0 | 98.5 | 93 | 108.6 | 99.98 | 104 | 95.5 |
| | 2 | 63.4 | 47 | 39.37 | 97.05 | 96.7 | 92.3 |
| | 4 | 36 | 18.1 | 29.67 | 86.95 | 81.1 | 78.7 |
| | 8 | 14.4 | 2.1 | 2.757 | 30.62 | 43.1 | 35.8 |

HCC1395

| Pictilisib | | | iCtrl | | | iMAPK4 | |
|---|---|---|---|---|---|---|---|
| | 0 | 104 | 101 | 95.69 | 97.98 | 98.8 | 103 |
| | 2 | 46.8 | 58.5 | 53.85 | 88.18 | 95 | 82.9 |
| | 4 | 3.15 | 4.41 | 3.09 | 41.43 | 44.6 | 47.4 |
| | 8 | 0.92 | 0 | 1.374 | 9.159 | 4.93 | 11.8 |

| Alpelisib | | | iCtrl | | | iMAPK4 | |
|---|---|---|---|---|---|---|---|
| | 0 | 104 | 101 | 95.69 | 97.98 | 98.8 | 103 |
| | 2 | 47.1 | 44.8 | 52.71 | 83.14 | 97 | 90.6 |
| | 4 | 4.86 | 5.84 | 5.265 | 44.83 | 51 | 41.1 |
| | 8 | 1.66 | 0 | 3.205 | 5.964 | 8.06 | 13.9 |

HCC1806

| Pictilisib | | | iCtrl | | | iMAPK4 | |
|---|---|---|---|---|---|---|---|
| | 0 | 89 | 101 | 109.8 | 106 | 101 | 92.5 |
| | 2 | 44.3 | 53.7 | 40.41 | 99.61 | 89.3 | 101 |
| | 4 | 15.9 | 20 | 11.93 | 30.26 | 31.8 | 29.9 |
| | 8 | 11.4 | 13.3 | 15.94 | 5.815 | 3.54 | 4.42 |

| Alpelisib | | | iCtrl | | | iMAPK4 | |
|---|---|---|---|---|---|---|---|
| | 0 | 89 | 101 | 109.8 | 106 | 101 | 92.5 |
| | 2 | 42.8 | 38.8 | 51.24 | 101.9 | 95.8 | 89.4 |
| | 4 | 25.4 | 23.2 | 13.27 | 34.63 | 32 | 28.1 |
| | 8 | 11 | 16.4 | 17.4 | 4.285 | 6.82 | 5.03 |

Source Data for supplementary Fig. 2a

| | iNT | | | iG2 | | | iG4 | | |
|---|---|---|---|---|---|---|---|---|---|
| 8h | 15.6 | 22.34 | 18.57 | 16.35 | 13.72 | 18.43 | 14.38 | 12.79 | 15.95 |
| 24h | 80.2 | 88.32 | 83.41 | 52.56 | 52.34 | 47.42 | 57.46 | 60.68 | 55.77 |

Source Data for supplementary Fig. 2b

| | WT | | | MAPK4 KO | | |
|---|---|---|---|---|---|---|
| 8h | 33.42 | 38.42 | 28.45 | 35.52 | 30.92 | 29.04 |
| 24h | 66.76 | 73.24 | 58.19 | 61.23 | 56.37 | 70.43 |

Source Data for supplementary Fig. 2c

| | iCtrl | | | iMAPK4 | | |
|---|---|---|---|---|---|---|
| 8h | 18.32 | 15.34 | 19.04 | 20.41 | 23.57 | 15.32 |
| 24h | 80.43 | 77.74 | 75.82 | 85.32 | 88.58 | 81.34 |

Source Data for supplementary Fig. 2d

| | iCtrl | | | iMAPK4 | | |
|---|---|---|---|---|---|---|
| 8h | 35.17 | 30.45 | 38.82 | 40.58 | 37.28 | 38.37 |
| 24h | 50.42 | 51.07 | 48.38 | 55.57 | 52.41 | 56.91 |
