# Supplementary material for: MAPK4 promotes triple negative breast cancer growth and reduces tumor sensitivity to PI3K blockade
Source: Nat Commun. 2022 Jan 11;13:245. doi: 10.1038/s41467-021-27921-1 (PMC8752662; doi:10.1038/s41467-021-27921-1)
Supplement: Supplementary file 3 — Reporting Summary [file 41467_2021_27921_MOESM3_ESM.pdf]

nature portfolio

Corresponding author(s): Feng Yang

Last updated by author(s): Dec 7, 2021

Reporting Summary

Nature Portfolio wishes to improve the reproducibility of the work that we publish. This form provides structure for consistency and transparency in reporting. For further information on Nature Portfolio policies, see our Editorial Policies and the Editorial Policy Checklist.

Statistics

For all statistical analyses, confirm that the following items are present in the figure legend, table legend, main text, or Methods section.

n/a	Confirmed	
☐	☒	The exact sample size (*n*) for each experimental group/condition, given as a discrete number and unit of measurement
☐	☒	A statement on whether measurements were taken from distinct samples or whether the same sample was measured repeatedly
☐	☒	The statistical test(s) used AND whether they are one- or two-sided<br>*Only common tests should be described solely by name; describe more complex techniques in the Methods section.*
☐	☒	A description of all covariates tested
☐	☒	A description of any assumptions or corrections, such as tests of normality and adjustment for multiple comparisons
☐	☒	A full description of the statistical parameters including central tendency (e.g. means) or other basic estimates (e.g. regression coefficient) AND variation (e.g. standard deviation) or associated estimates of uncertainty (e.g. confidence intervals)
☐	☒	For null hypothesis testing, the test statistic (e.g. *F*, *t*, *r*) with confidence intervals, effect sizes, degrees of freedom and *P* value noted<br>*Give P values as exact values whenever suitable.*
☒	☐	For Bayesian analysis, information on the choice of priors and Markov chain Monte Carlo settings
☒	☐	For hierarchical and complex designs, identification of the appropriate level for tests and full reporting of outcomes
☒	☐	Estimates of effect sizes (e.g. Cohen's *d*, Pearson's *r*), indicating how they were calculated

Our web collection on statistics for biologists contains articles on many of the points above.

Software and code

Policy information about availability of computer code

Data collection	*Provide a description of all commercial, open source and custom code used to collect the data in this study, specifying the version used OR state that no software was used.*
Data analysis	GraphPad Prism software, 9.3

For manuscripts utilizing custom algorithms or software that are central to the research but not yet described in published literature, software must be made available to editors and reviewers. We strongly encourage code deposition in a community repository (e.g. GitHub). See the Nature Portfolio guidelines for submitting code & software for further information.

Data

Policy information about availability of data

All manuscripts must include a data availability statement. This statement should provide the following information, where applicable:

- Accession codes, unique identifiers, or web links for publicly available datasets
- A description of any restrictions on data availability
- For clinical datasets or third party data, please ensure that the statement adheres to our policy

Source data are provided with this paper as a Source Data file.

Field-specific reporting

Please select the one below that is the best fit for your research. If you are not sure, read the appropriate sections before making your selection.

- [x] Life sciences
- [ ] Behavioural & social sciences
- [ ] Ecological, evolutionary & environmental sciences

For a reference copy of the document with all sections, see nature.com/documents/nr-reporting-summary-flat.pdf

Life sciences study design

All studies must disclose on these points even when the disclosure is negative.

Sample size	Sample sizes were determined based on our previous experience of similar studies (JCI, 2019, PMID: 30688659; JCI, 2021 PMID: 33586682; Science Advances, 2021, PMID: 34767444).
Data exclusions	No data were excluded from the analyses.
Replication	We performed biologically independent replications of all studies. The attempts were successful. When there were notable experimental variations, we performed additional biologically independent replication studies and made sure that the reported results were reproduced in three consecutive studies.
Randomization	The samples, including those in cell culture studies in vitro and xenograft studies in Figure 10 were randomly allocated into experimental vs. control groups.
Blinding	Since most studies were performed by individual researcher knowing the design of the studies, blinding data collection and analysis was not performed.

Reporting for specific materials, systems and methods

We require information from authors about some types of materials, experimental systems and methods used in many studies. Here, indicate whether each material, system or method listed is relevant to your study. If you are not sure if a list item applies to your research, read the appropriate section before selecting a response.

Materials & experimental systems

n/a	Involved in the study	
[ ]	[x]	Antibodies
[ ]	[x]	Eukaryotic cell lines
[x]	[ ]	Palaeontology and archaeology
[ ]	[x]	Animals and other organisms
[x]	[ ]	Human research participants
[x]	[ ]	Clinical data
[x]	[ ]	Dual use research of concern

Methods

n/a	Involved in the study	
[x]	[ ]	ChIP-seq
[x]	[ ]	Flow cytometry
[x]	[ ]	MRI-based neuroimaging

Antibodies

Antibodies used	anti-p-AKT T308 (Catalog 13038), p-AKT S473 (catalog 4060), AKT (Catalog 9272), and p-GSK3β S9 (Catalog 9336) from Cell Signaling Technology, anti-DYKDDDDK (Agilent, Catalog 200474), anti-MAPK4 (Abcepta, Catalog AP7298b), anti-BrdU (MilliporeSigma, Catalog B2531), anti-β-ACTIN (Abclonal, Catalog AC026 or MilliporeSigma, Catalog A1978). Dilutions: 1:5000 for anti-β-ACTIN antibody, 1:1000 for all others.
Validation	Most of these antibodies are widely used and have been published/validated by many research groups. The anti-β-ACTIN (Abclonal, Catalog AC0260) produced the same pattern as anti-β-ACTIN (MilliporeSigma, Catalog A1978), which is widely used.

Eukaryotic cell lines

Policy information about cell lines

Cell line source(s)	SUM159, MDA-MB-468, MDA-MB-231, HCC1395, HCC1806, HCC1937, MCF10A, and HS578T cells were from ATCC through a local core/lab.
Authentication	Acquired directly from a local core/lab with limited expansion; no further authentication was done.
Mycoplasma contamination	Free of contamination

Commonly misidentified lines (See ICLAC register)	No commonly misidentified cell lines were used in the study.

Animals and other organisms

Policy information about studies involving animals; ARRIVE guidelines recommended for reporting animal research

Laboratory animals	Female SCID/beige mice at 8-10 weeks old from Envigo were used in the xenograft studies. Mice were housed in a pathogen-free facility at Baylor College of Medicine. pathogen-free animal housing facility under regular 12-hour light/dark cycle at ambient room temperature and humidity.
Wild animals	No wild animals were used in the study.
Field-collected samples	No field-collected samples were used in the study,
Ethics oversight	Institutional Animal Care and Use Committee (IACUC) of Baylor College of Medicine.

Note that full information on the approval of the study protocol must also be provided in the manuscript.
