# Supplementary material for: MAPK4 promotes triple negative breast cancer growth and reduces tumor sensitivity to PI3K blockade
Source: Nat Commun. 2022 Jan 11;13:245. doi: 10.1038/s41467-021-27921-1 (PMC8752662; doi:10.1038/s41467-021-27921-1)

Figure 2 A

MAPK4 ABGENT (#7298b)

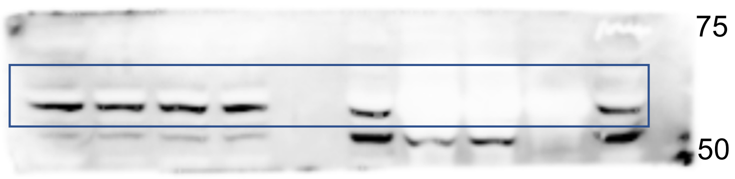

β actin Abclonal (AC026)

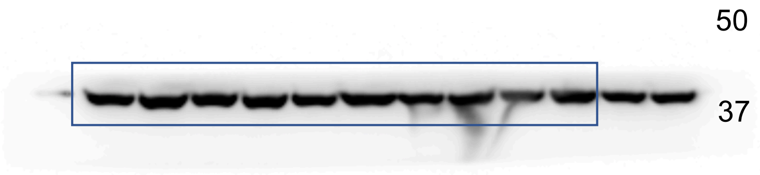

Figure 2B

MDA-MB-231

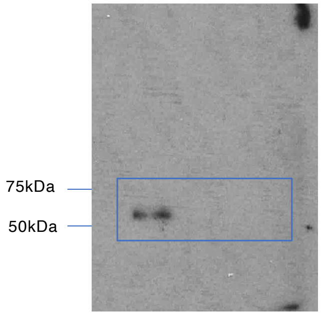

MAPK4
ABGENT (#7298b)

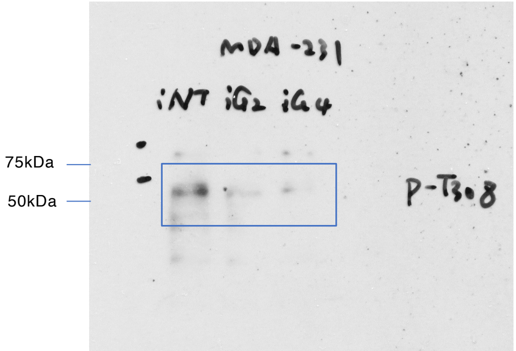

p-Akt T308 CST #13038

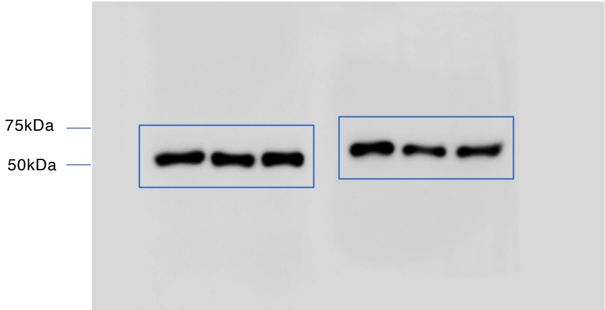

CST#9272 Akt p-Akt S473 CST #4060

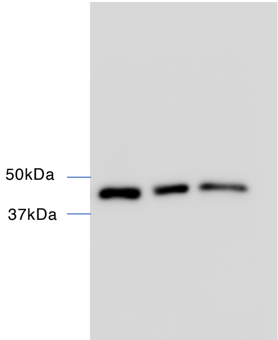

p-GSK3β S9 CST #9336

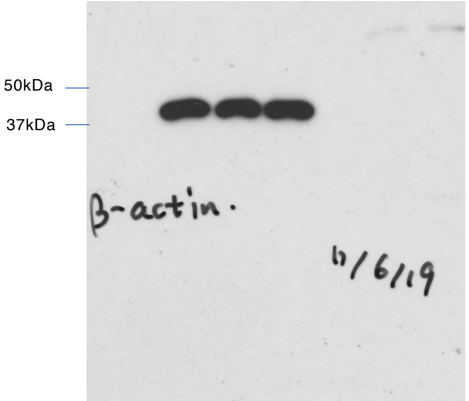

β-ACTIN Sigma, A1978

Figure 2B HCC1937

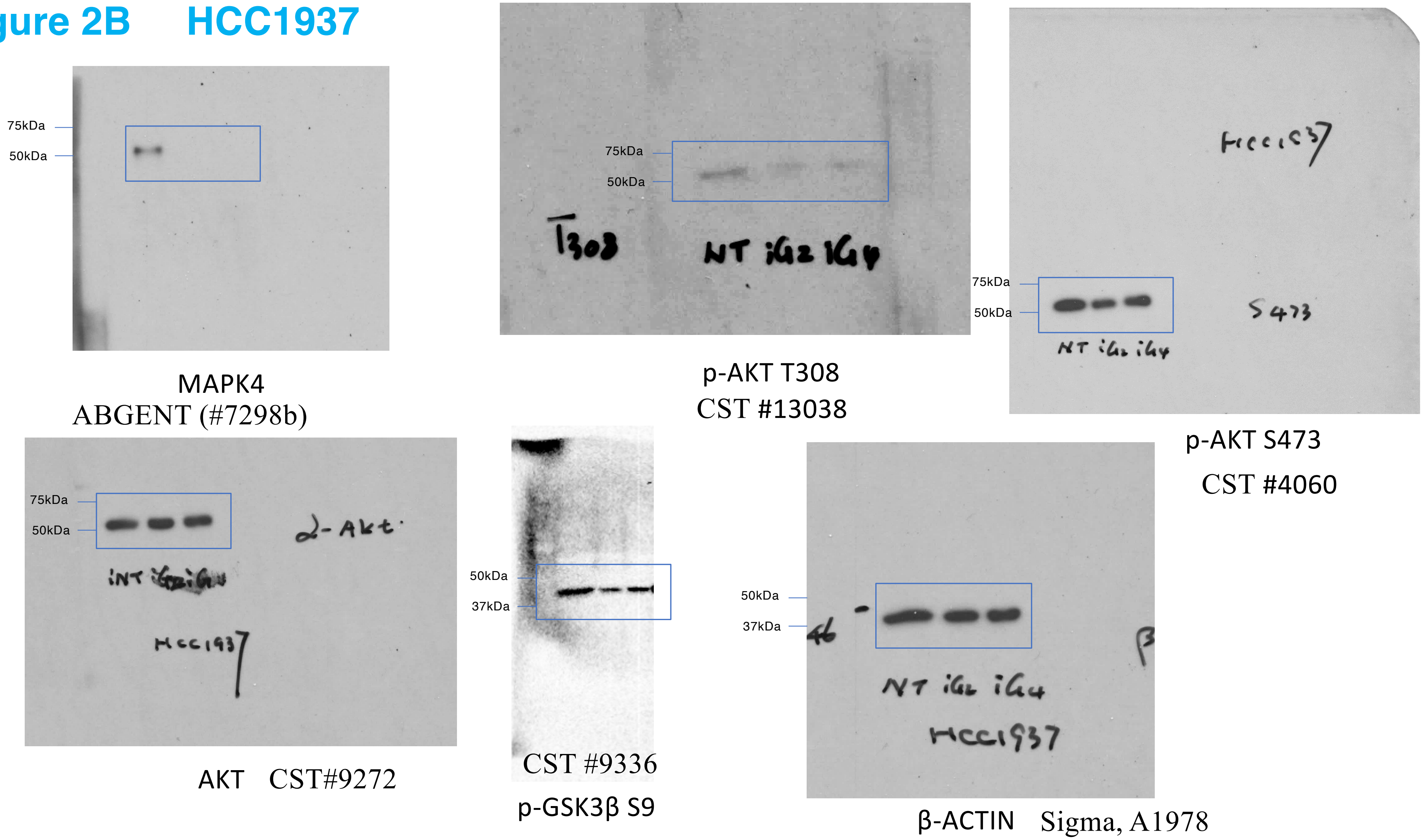

Figure 2 B HS578T

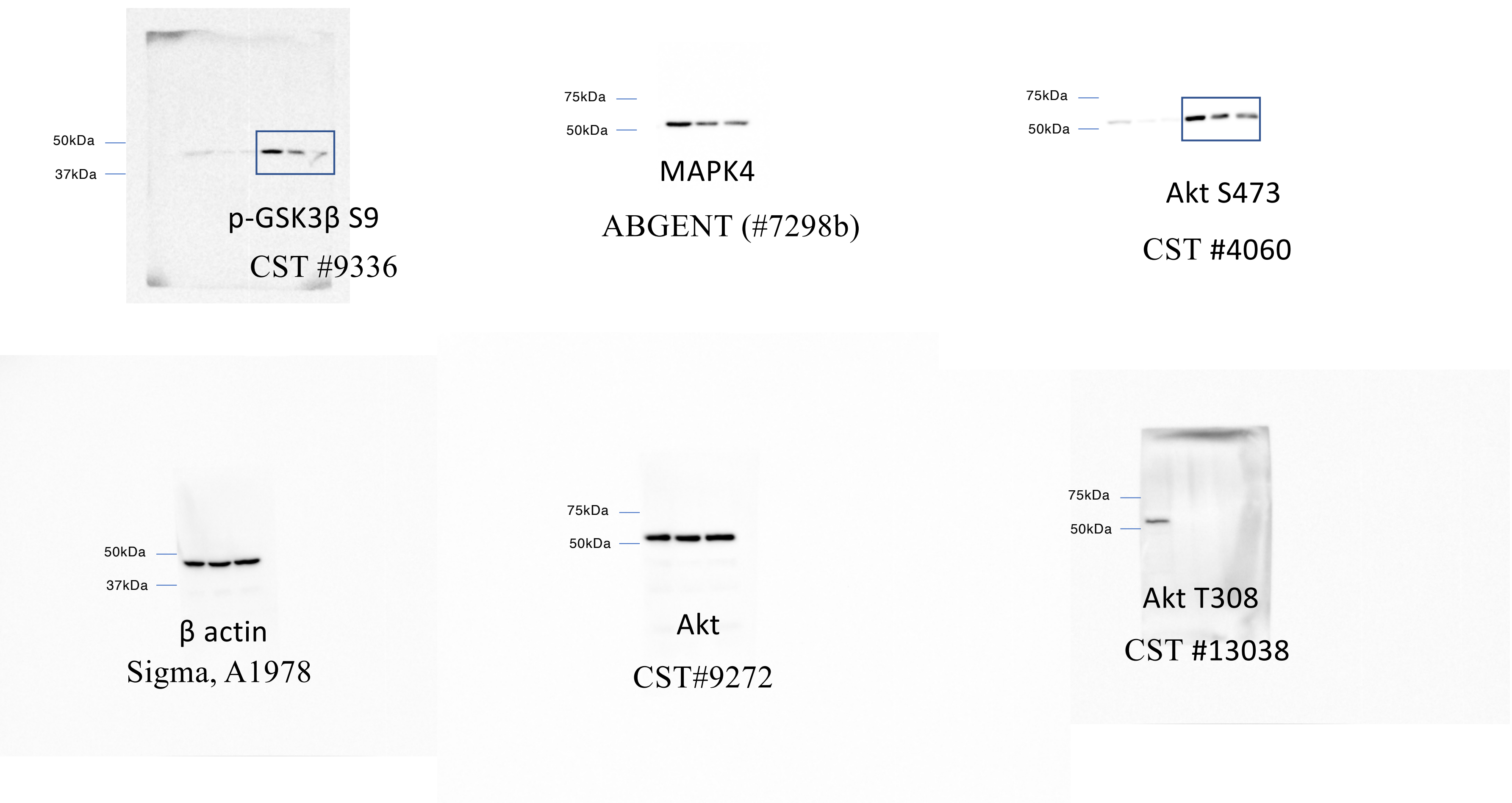

Figure 2 B SUM159

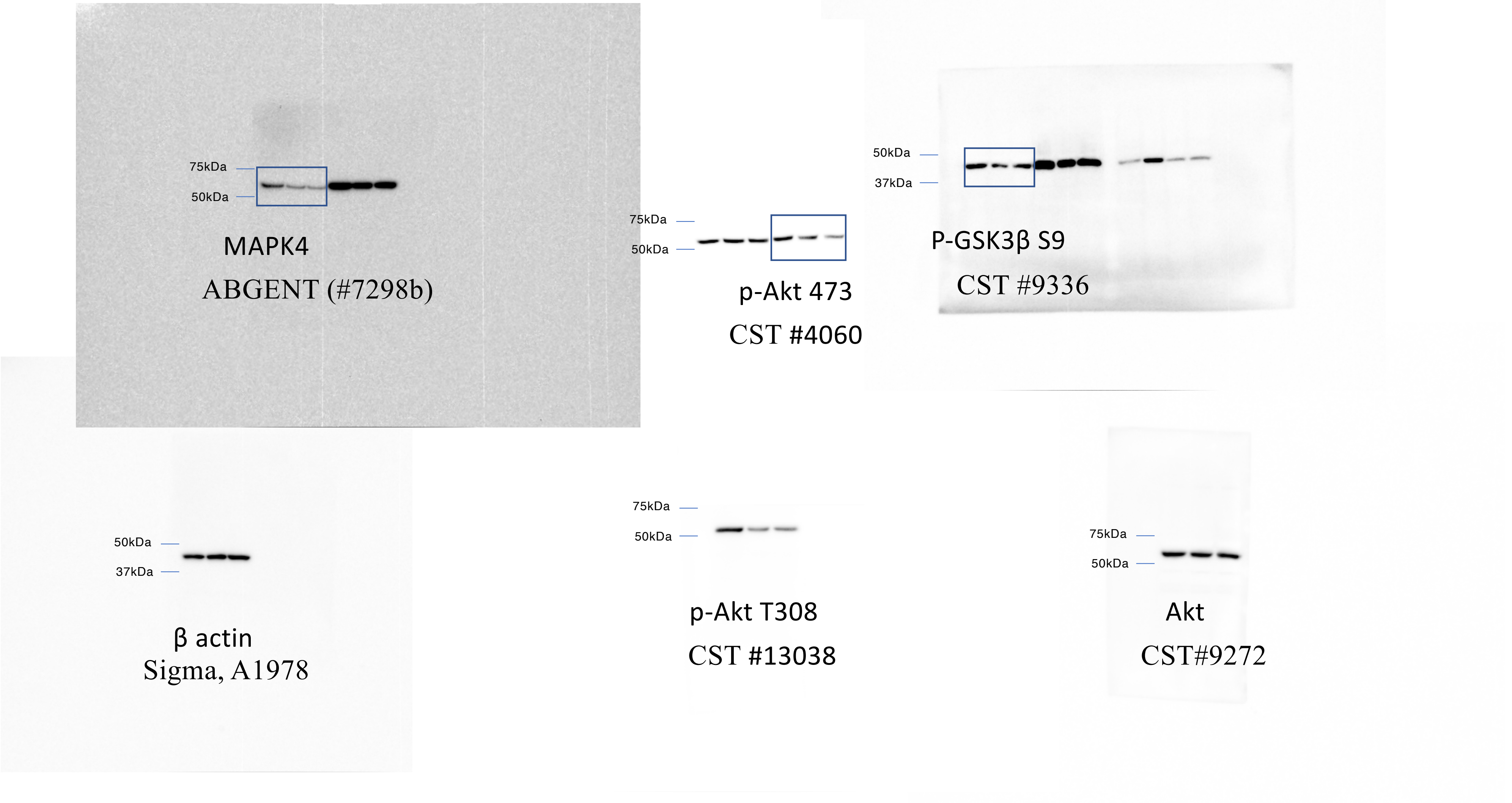

Figure 2 C SUM159

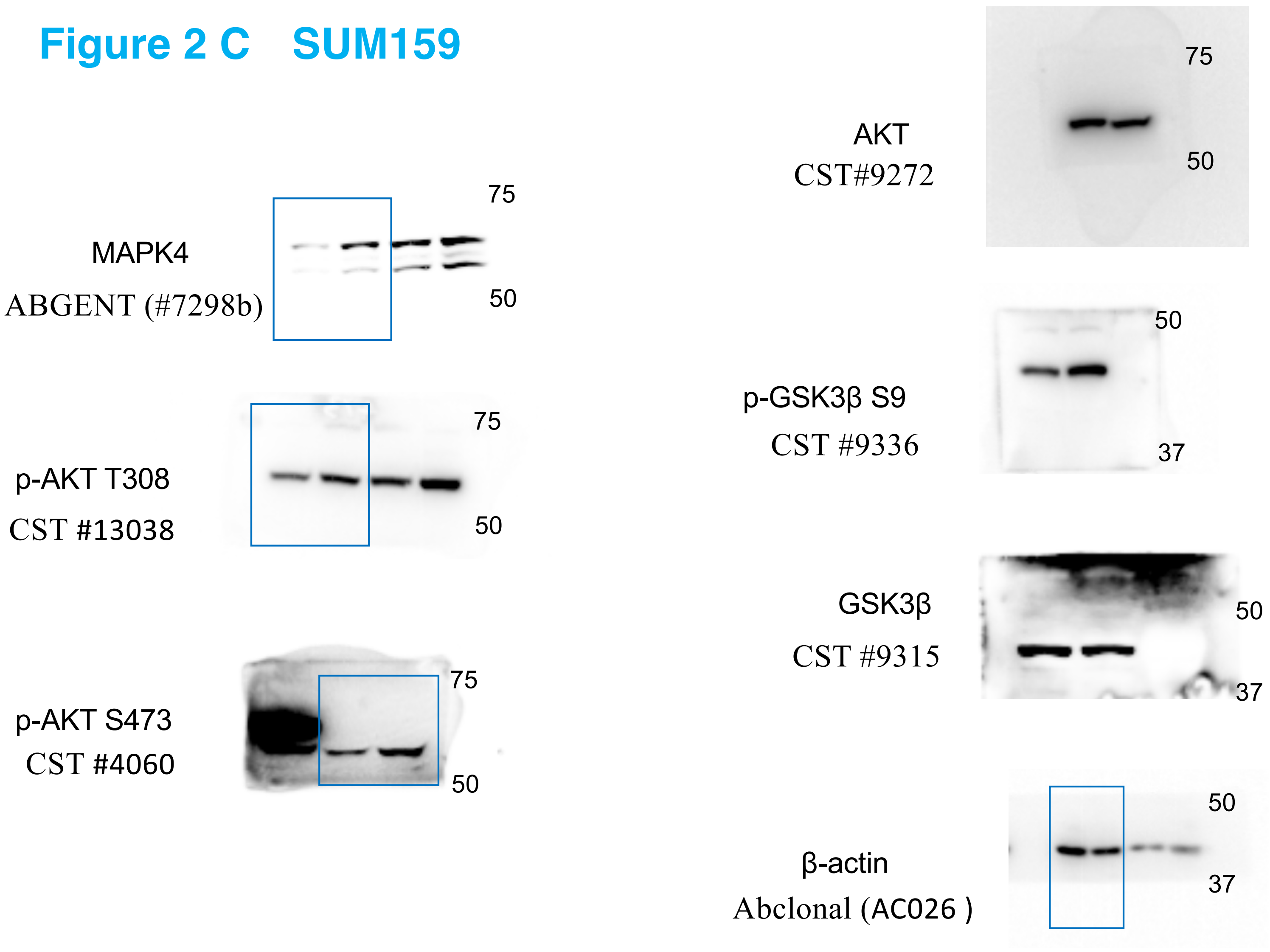

Figure 2 C MDA-MB-468

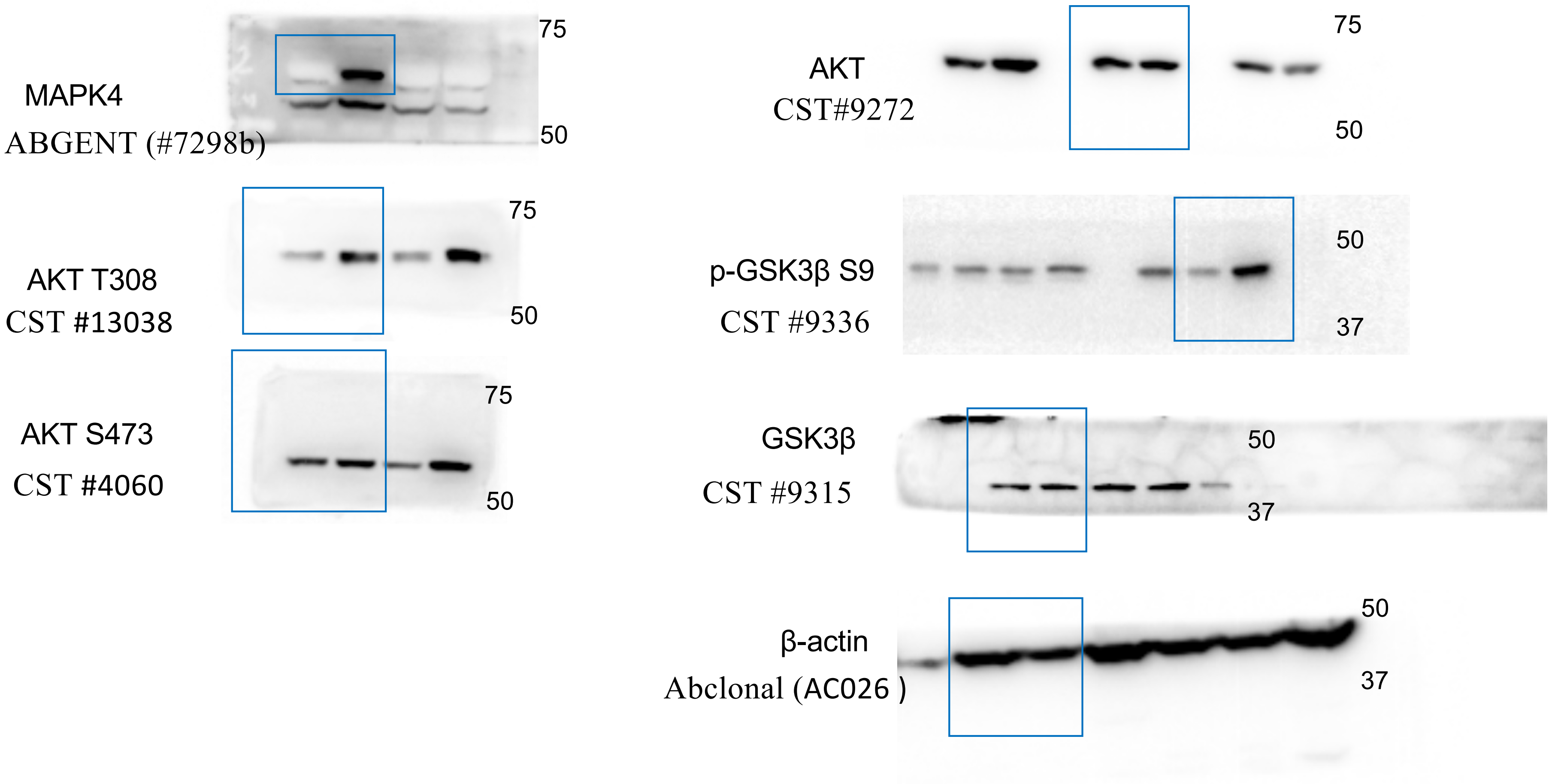

Figure 2 C HCC1395

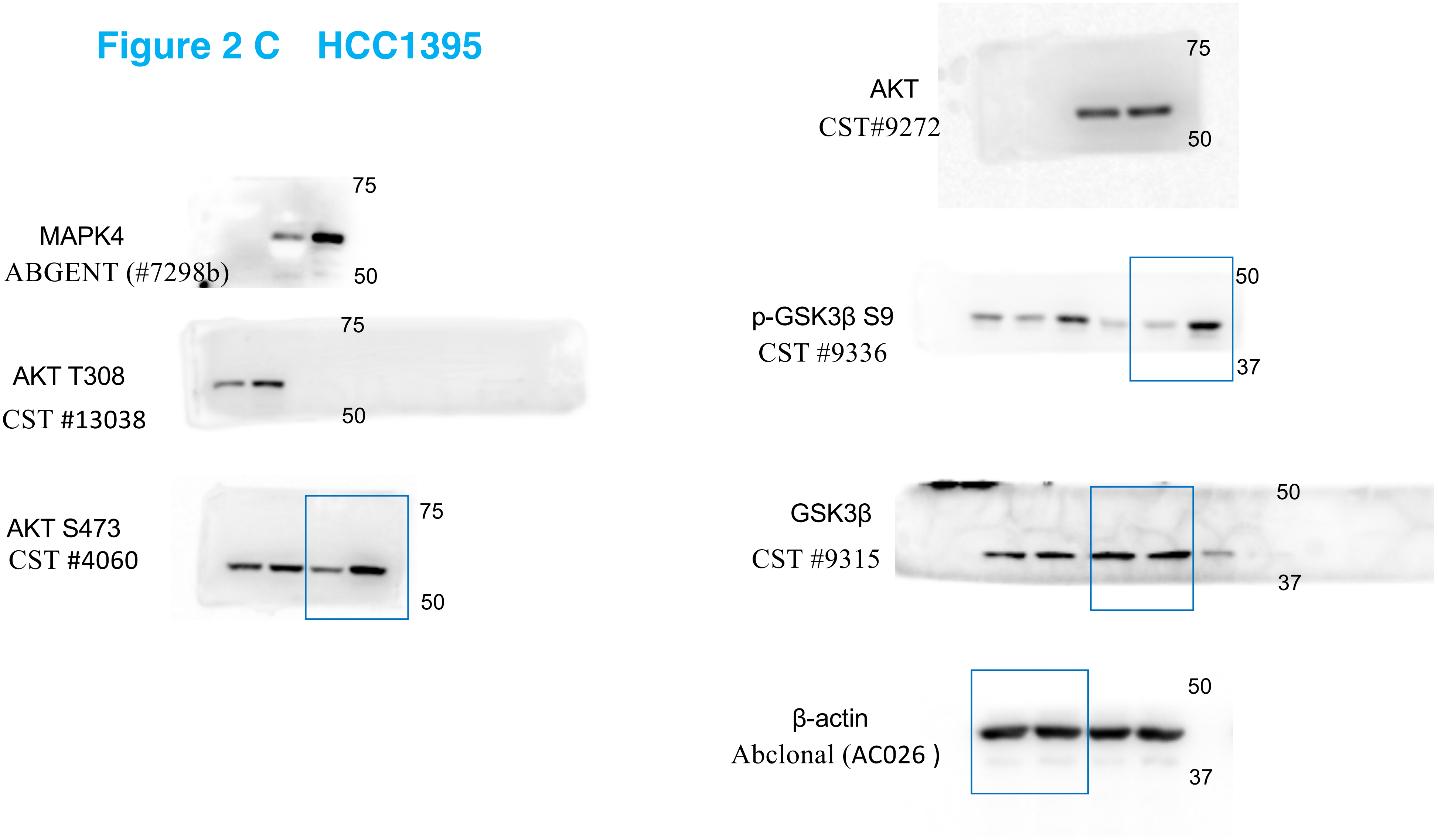

Figure 2 C HCC1806

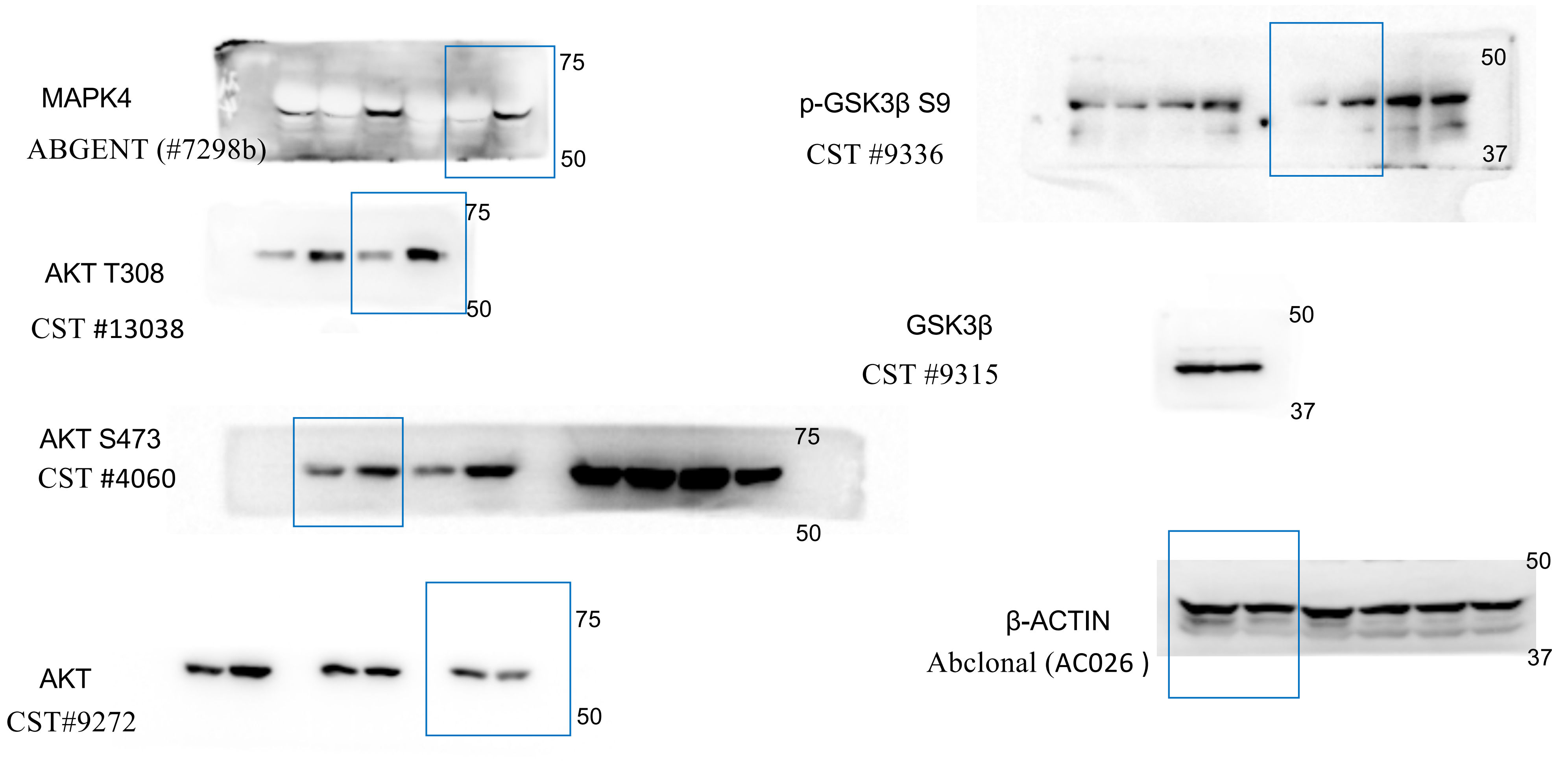

Figure 2 D MDA-MB-231

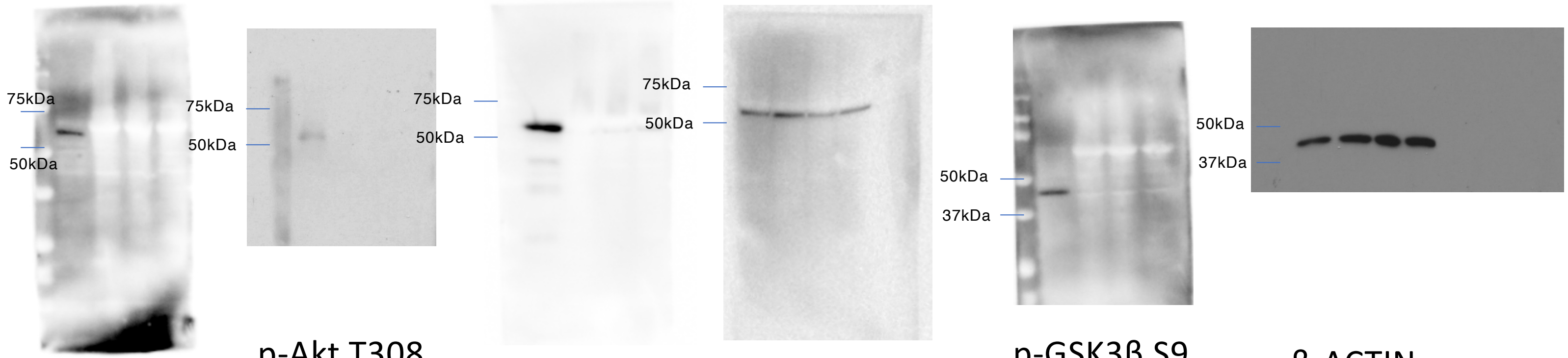

MAPK4
ABGENT (#7298b)

p-Akt T308
CST #13038

p-Akt S473
CST #4060

Akt
CST#9272

p-GSK3β S9
CST #9336

β-ACTIN
Sigma, A1978

Figure 2D SUM159

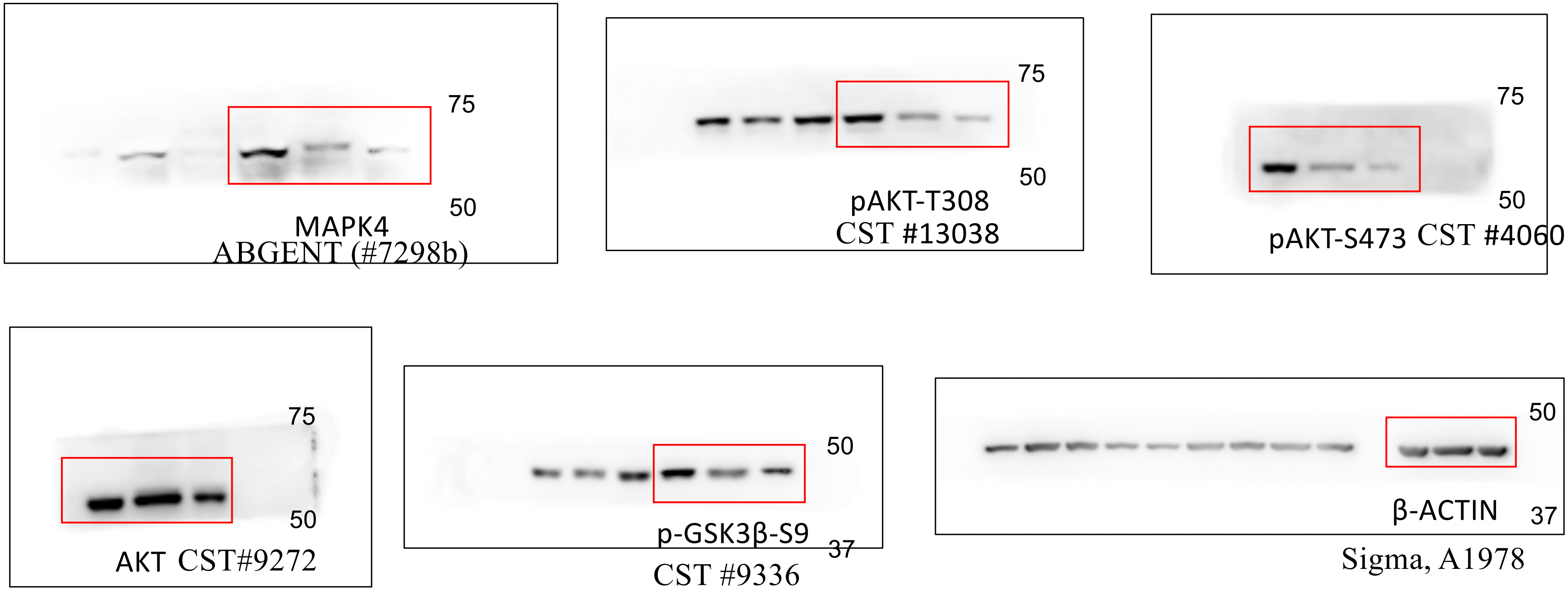

Figure 2E MDA-MB-231

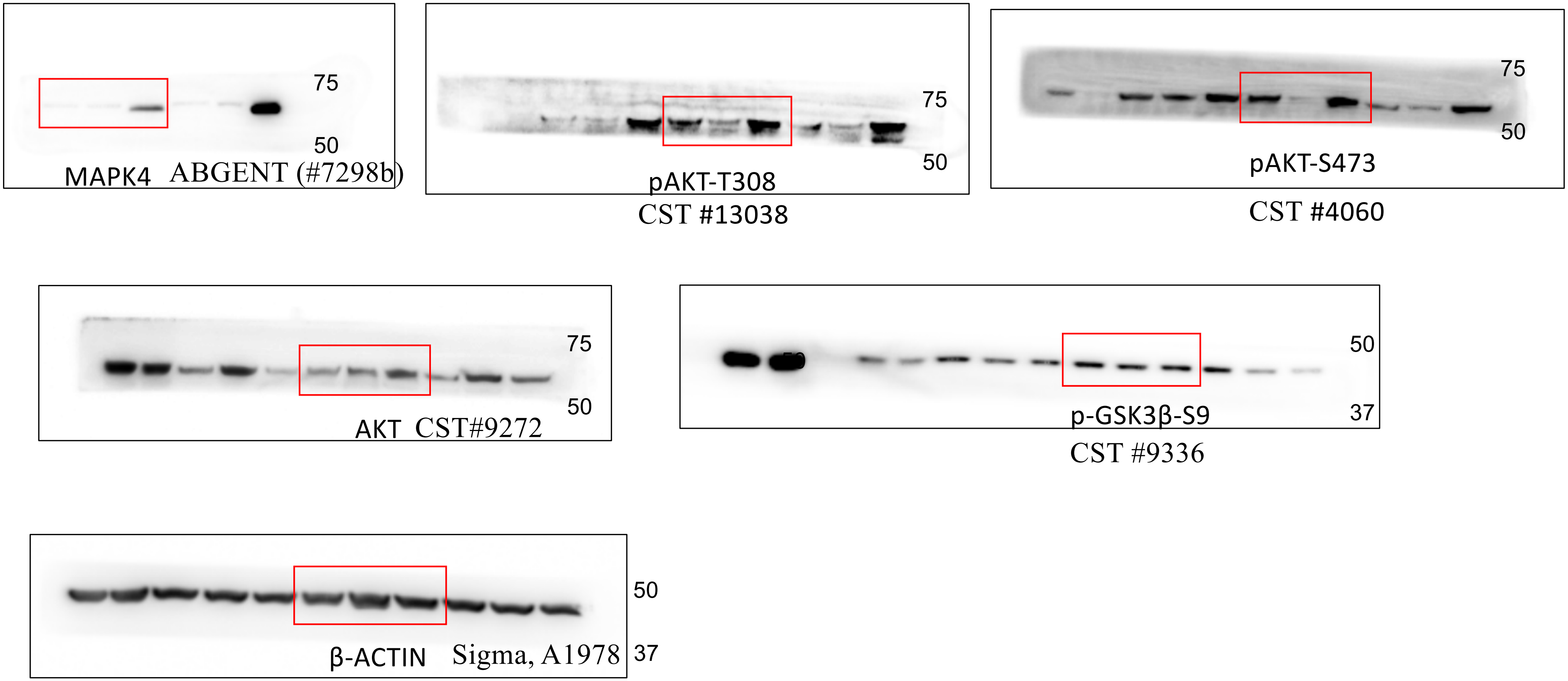

Figure 2E SUM159

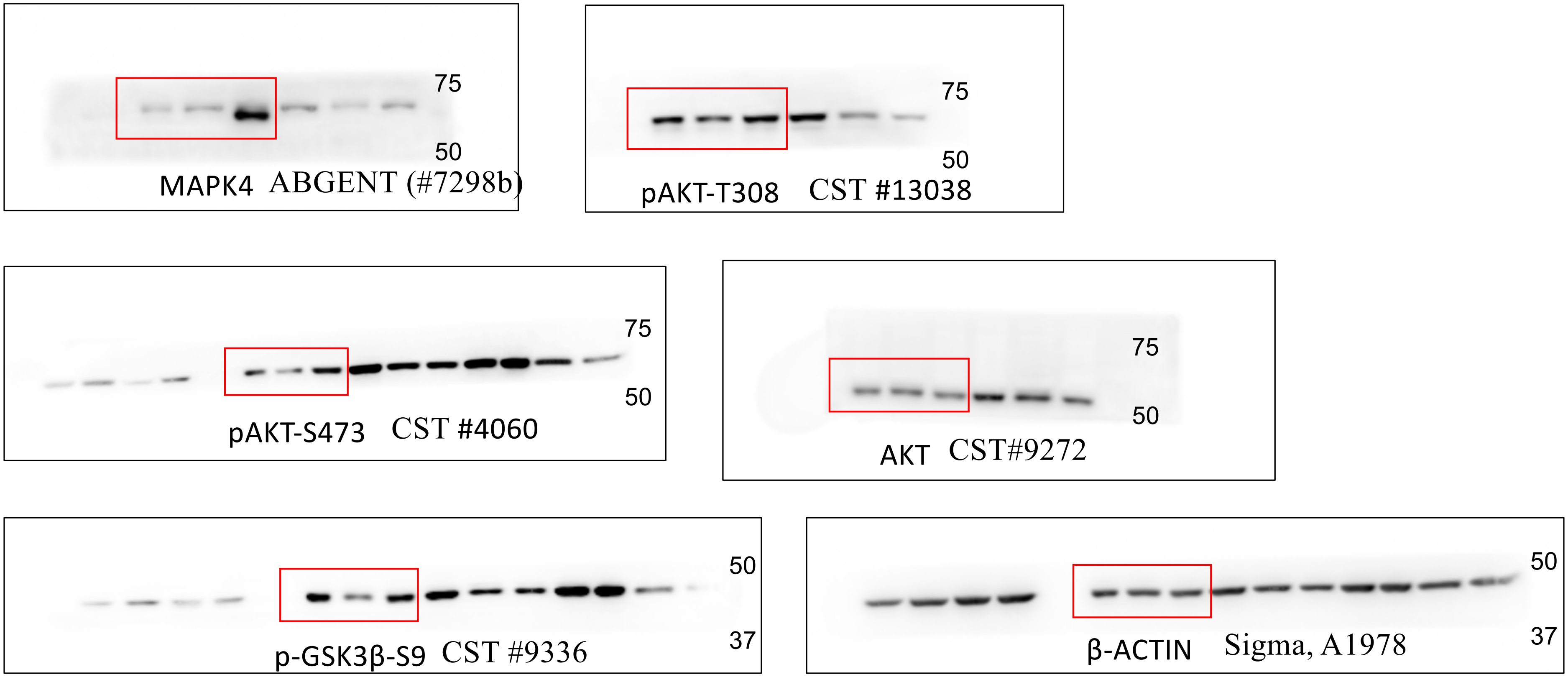

Figure 4 D

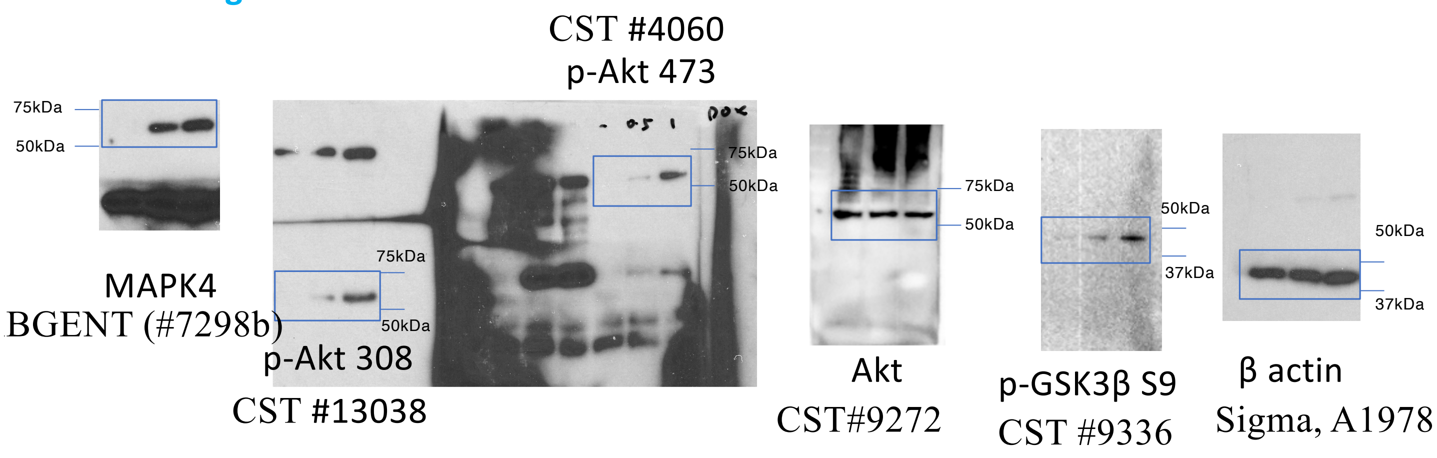
CST #4060
p-Akt 473
75kDa
50kDa
MAPK4
BGENT (#7298b)
75kDa
50kDa
p-Akt 308
CST #13038
75kDa
50kDa
Akt
CST#9272
50kDa
37kDa
p-GSK3β S9
CST #9336
50kDa
37kDa
β actin
Sigma, A1978

Figure 5A **MDA-MB-231**

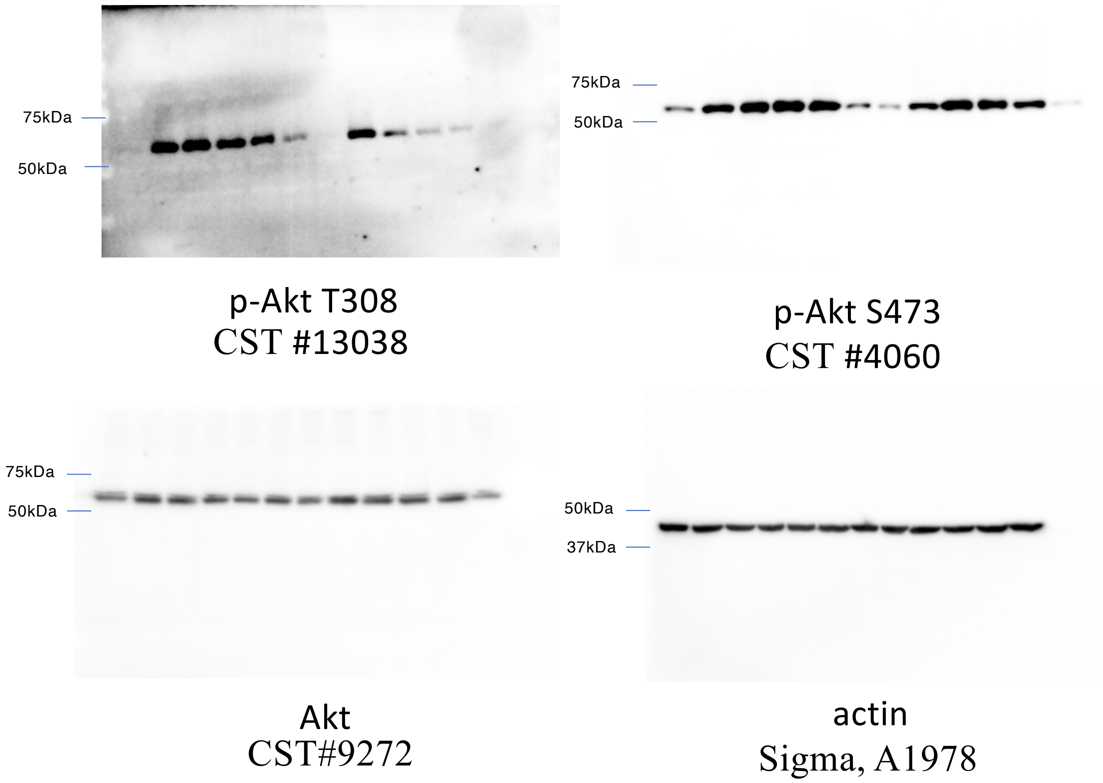

Figure 5A **HS578T**

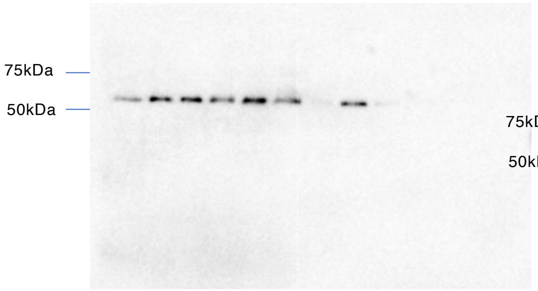

p-Akt T308
CST #13038

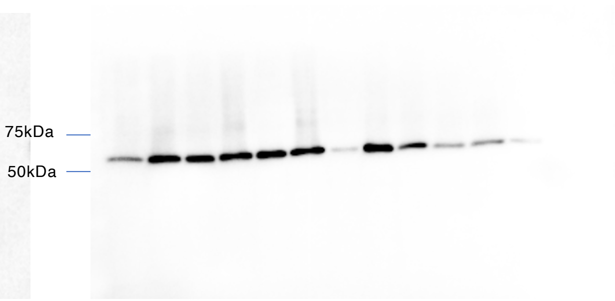

p-Akt S473
CST #4060

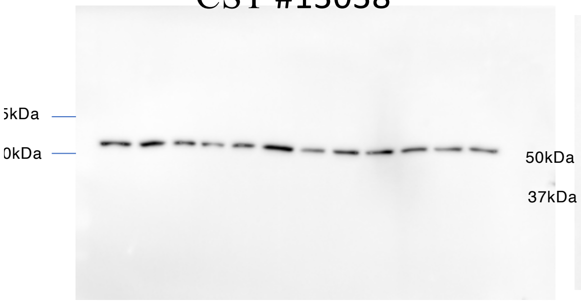

Akt
CST#9272

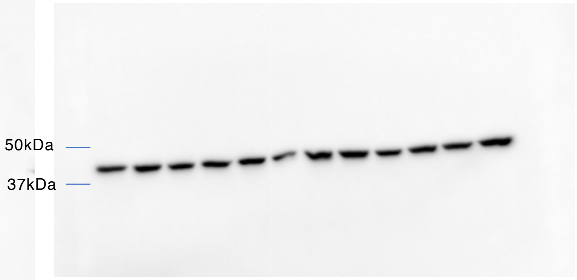

actin
Sigma, A1978

Figure 5B

SUM159 iNT/ishMAPK4 Pictilicib 20nM

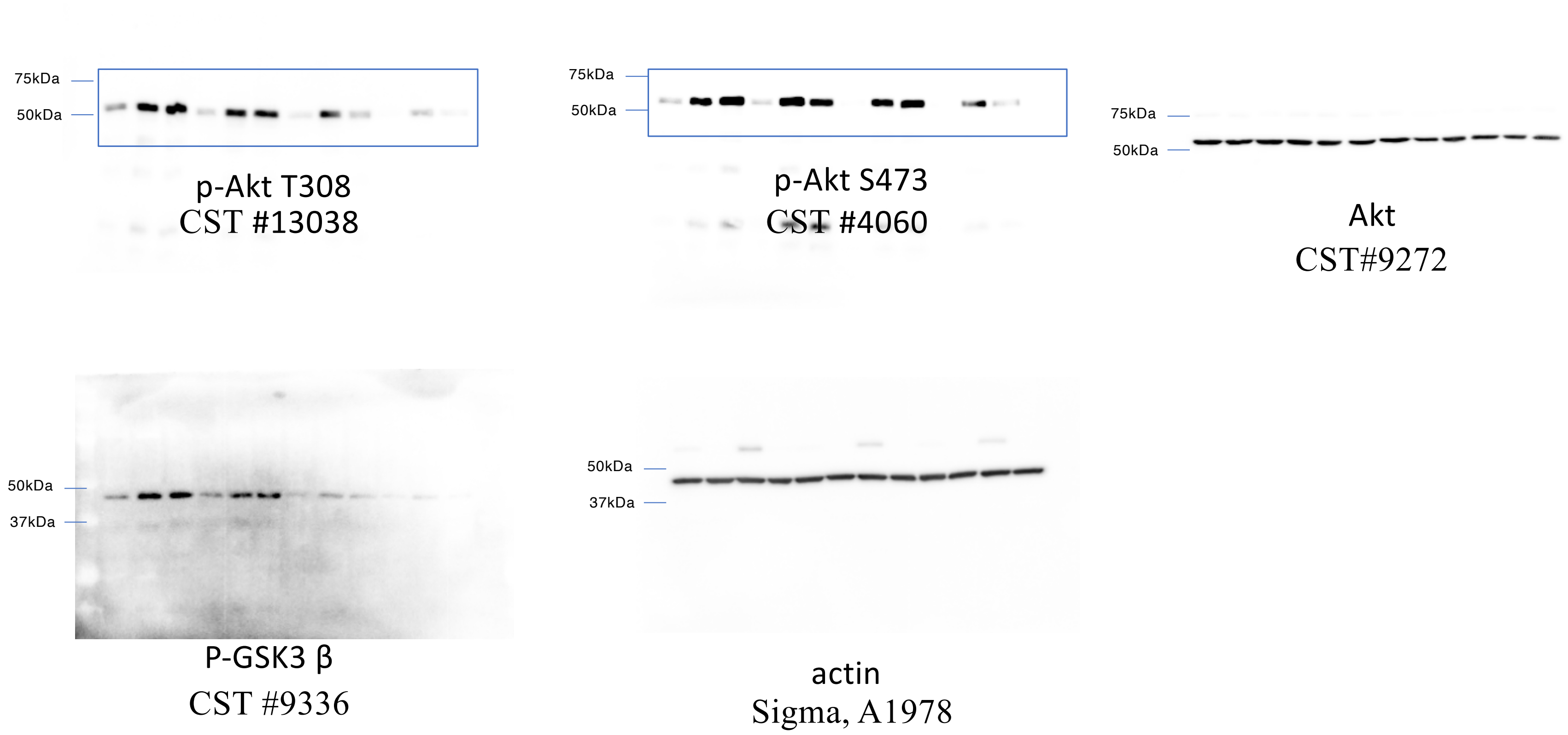

Figure 5B

SUM159 iNT/ishMAPK4 Alpelicib 100nM

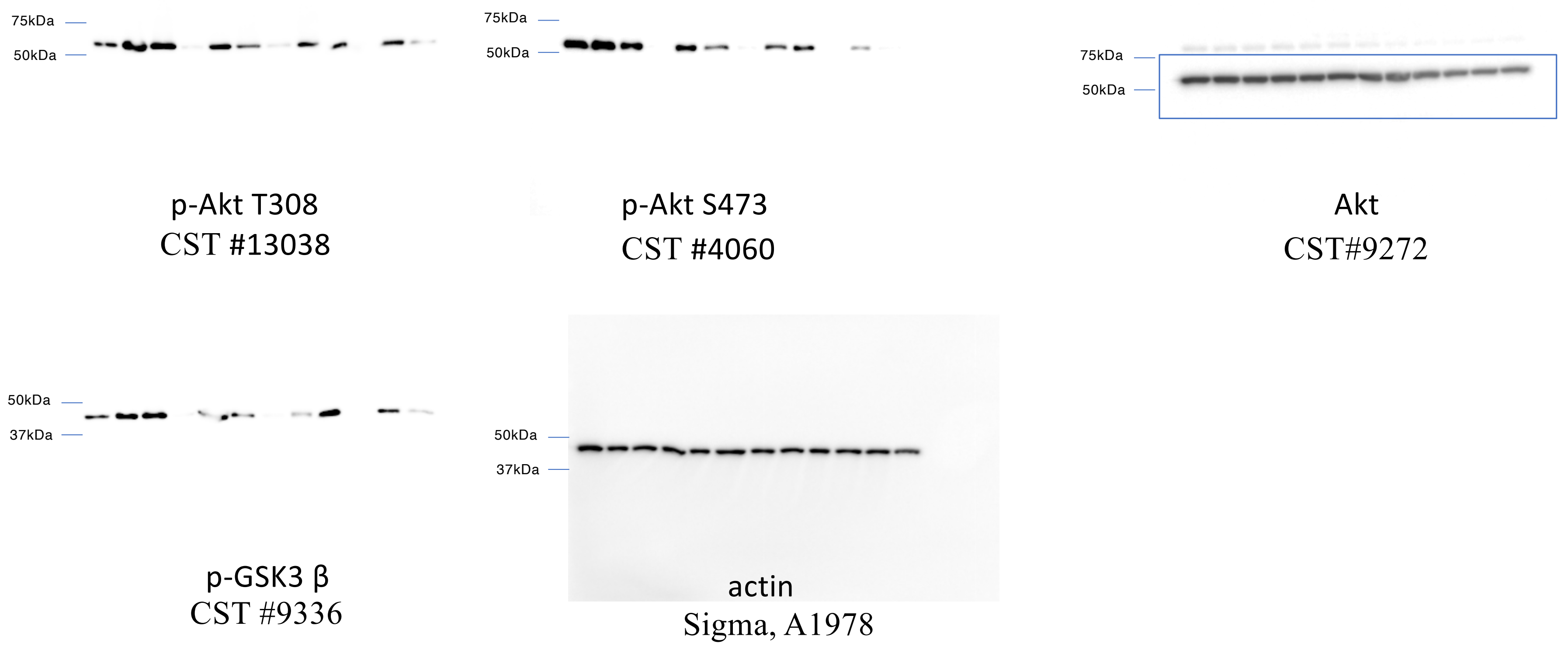

Figure 5C **MDA-MB-231 Pictilicib**

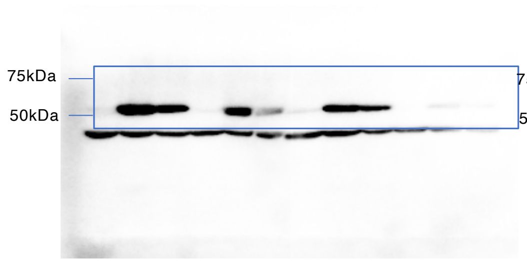

p-Akt T308
CST #13038

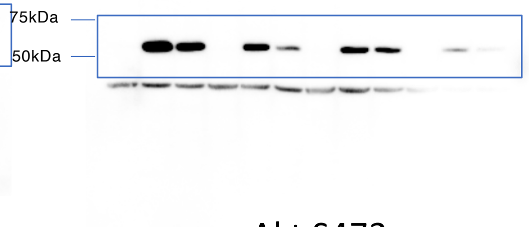

p-Akt S473
CST #4060

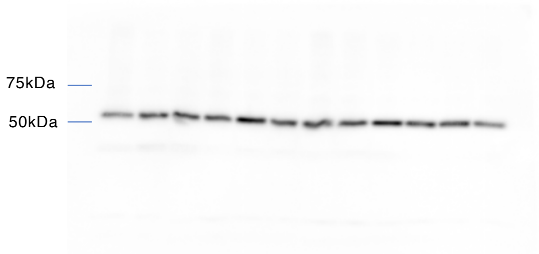

Akt
CST#9272

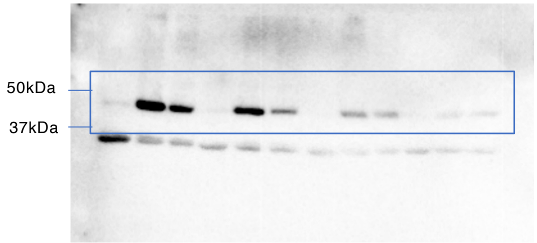

P-GSK3 β
CST #9336

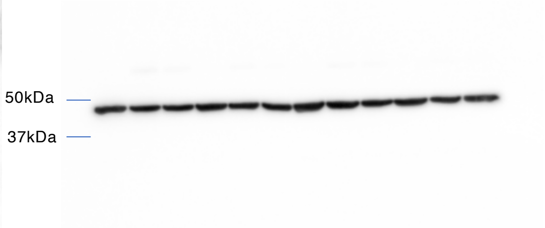

actin
Sigma, A1978

Figure 5C MDA-MB-231 Alpelicib

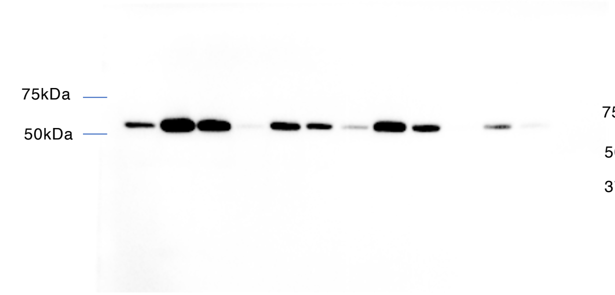

p-Akt T308
CST #13038

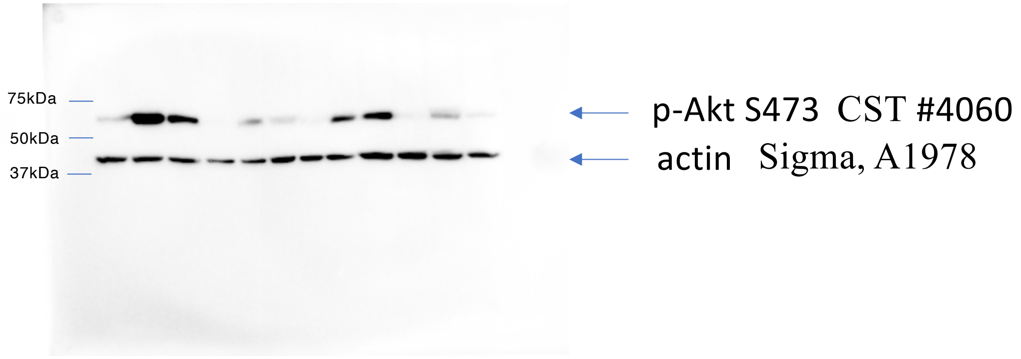

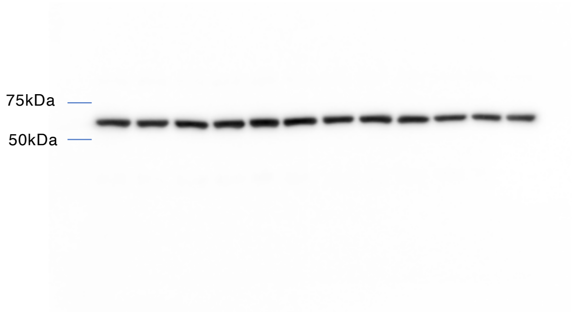

Akt
CST#9272

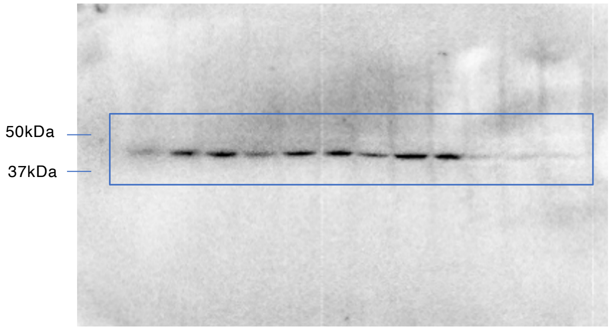

P-GSK3 β
CST #9336

Figure 5C   **SUM159 pictilisib**

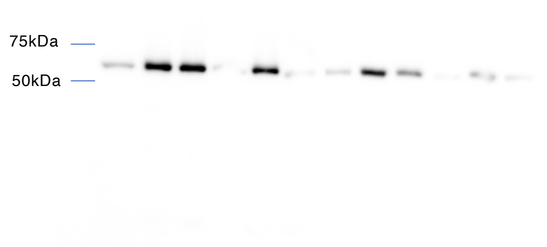

p-Akt T308   CST #13038

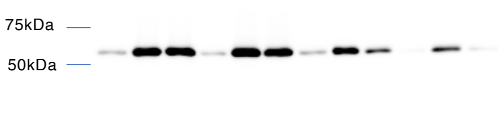

p-Akt S473
CST #4060

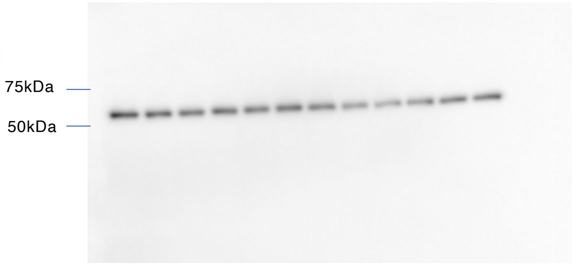

Akt   CST#9272

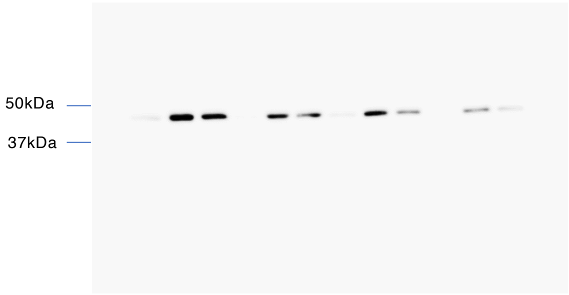

P-GSK3 β
CST #9336

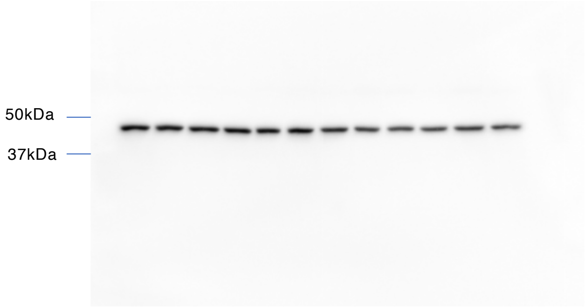

actin
Sigma, A1978

Figure 5C **SUM159 Alpelicib**

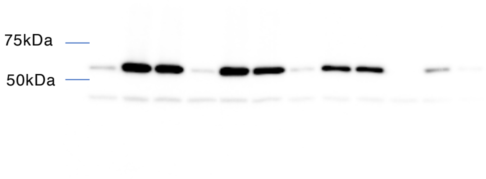

p-Akt T308
CST #13038

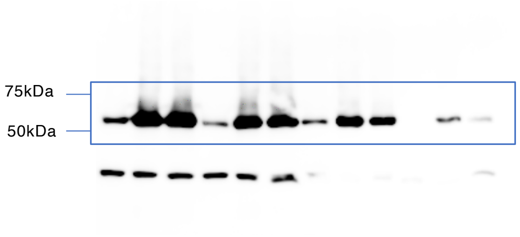

p-Akt S473
CST #4060

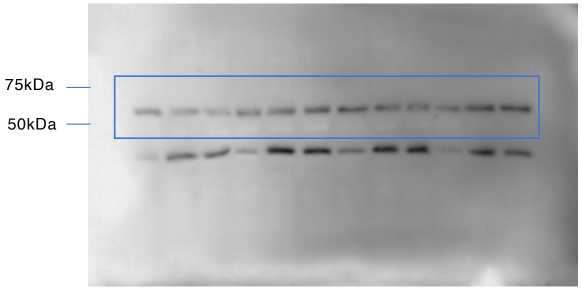

Akt
CST#9272

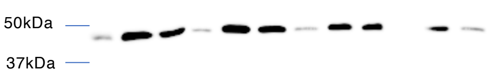

p-GSK3 β S9
CST #9336

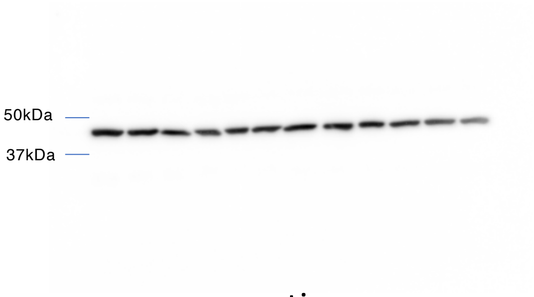

actin
Sigma, A1978

Figure 7F, SUM159 cells

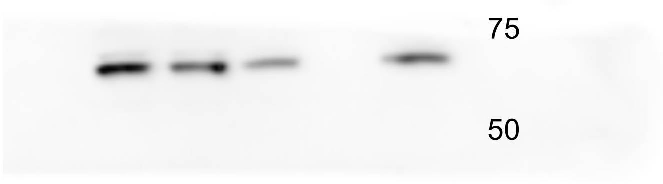

p-Akt T308
CST #13038

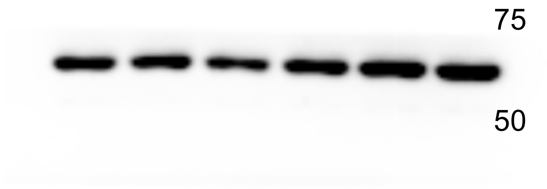

Akt
CST#9272

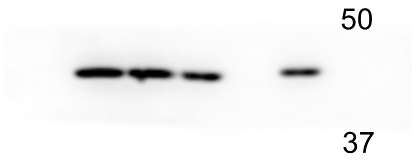

p-GSK3 β
CST #9336

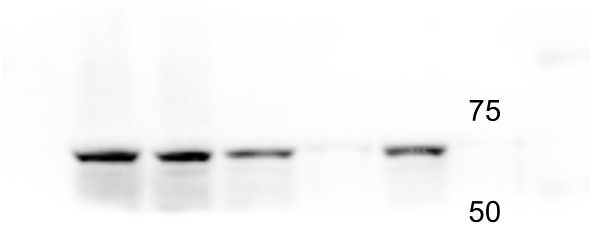

p-Akt S473
CST #4060

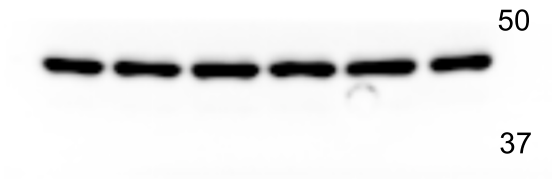

β-ACTIN
Sigma, A1978

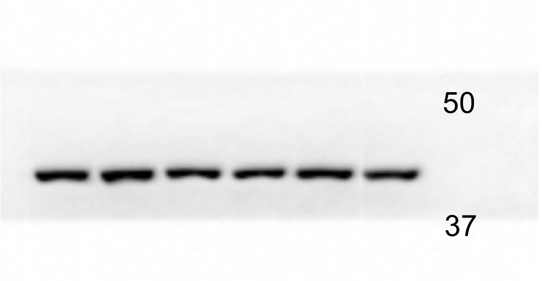

GSK3β
CST #9315

Figure 7F, MDA-MB-231 cells

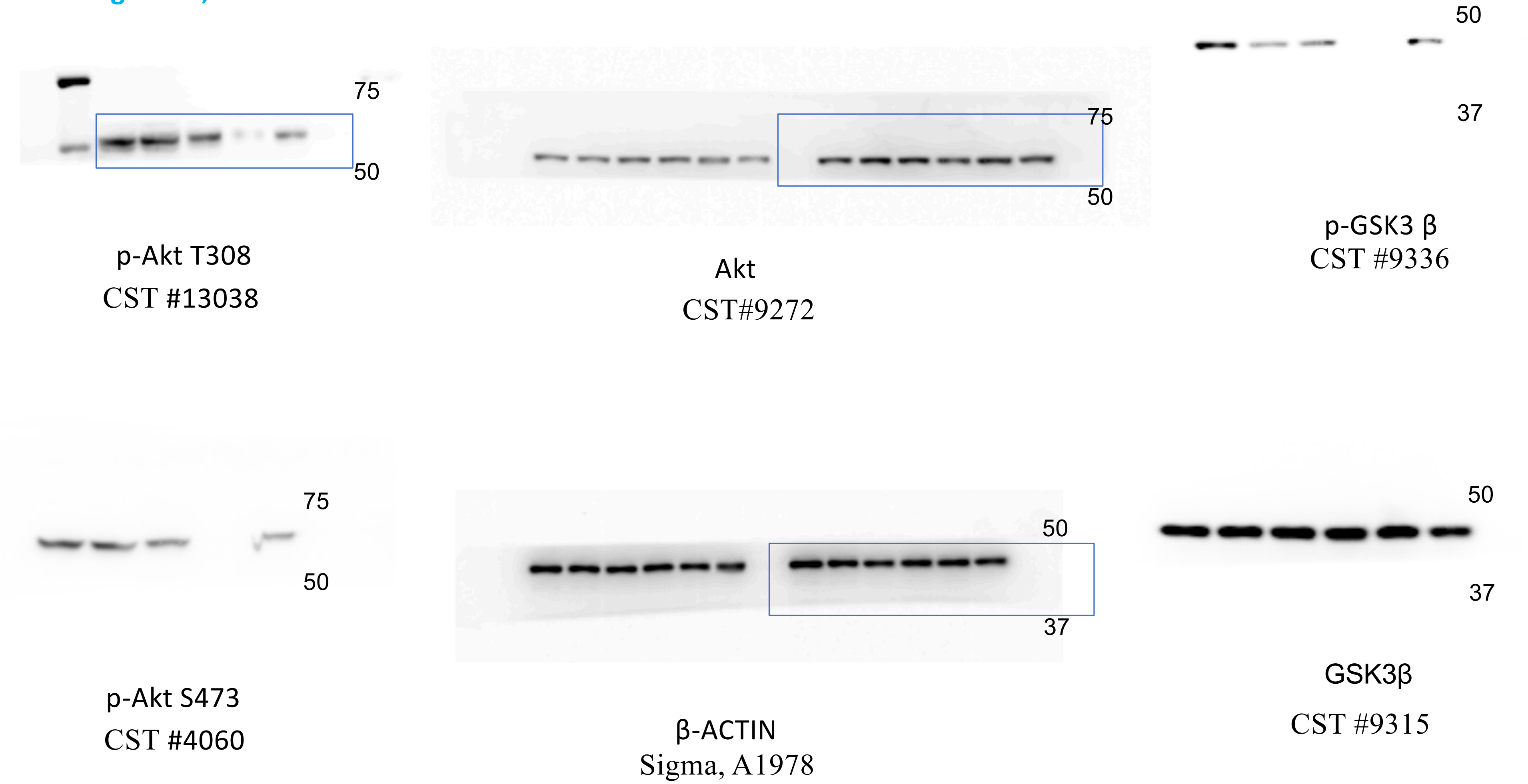

Figure 8E, SUM159 cells

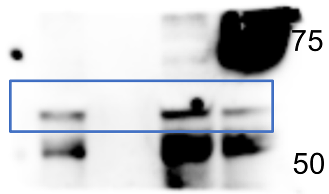

p-Akt T308
CST #13038

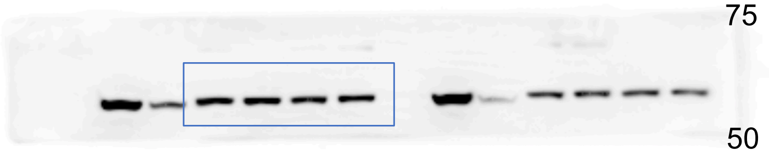

Akt
CST#9272

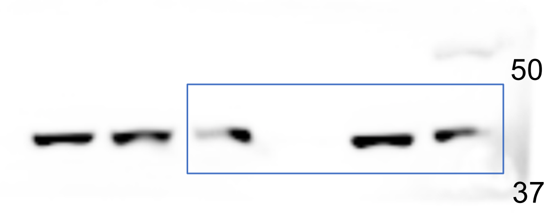

p-GSK3 β
CST #9336

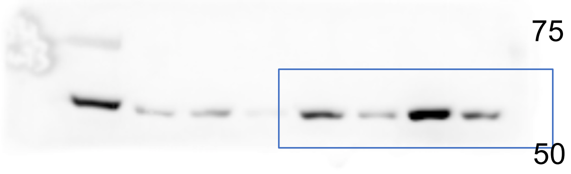

p-Akt S473
CST #4060

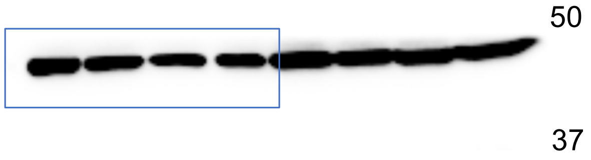

β-ACTIN
Sigma, A1978

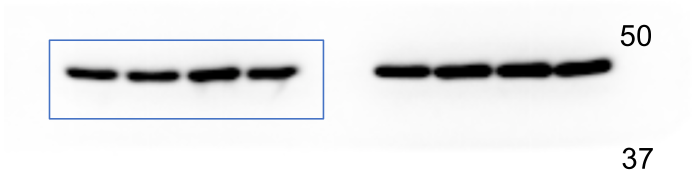

GSK3β
CST #9315

Figure 8E,   HCC1806

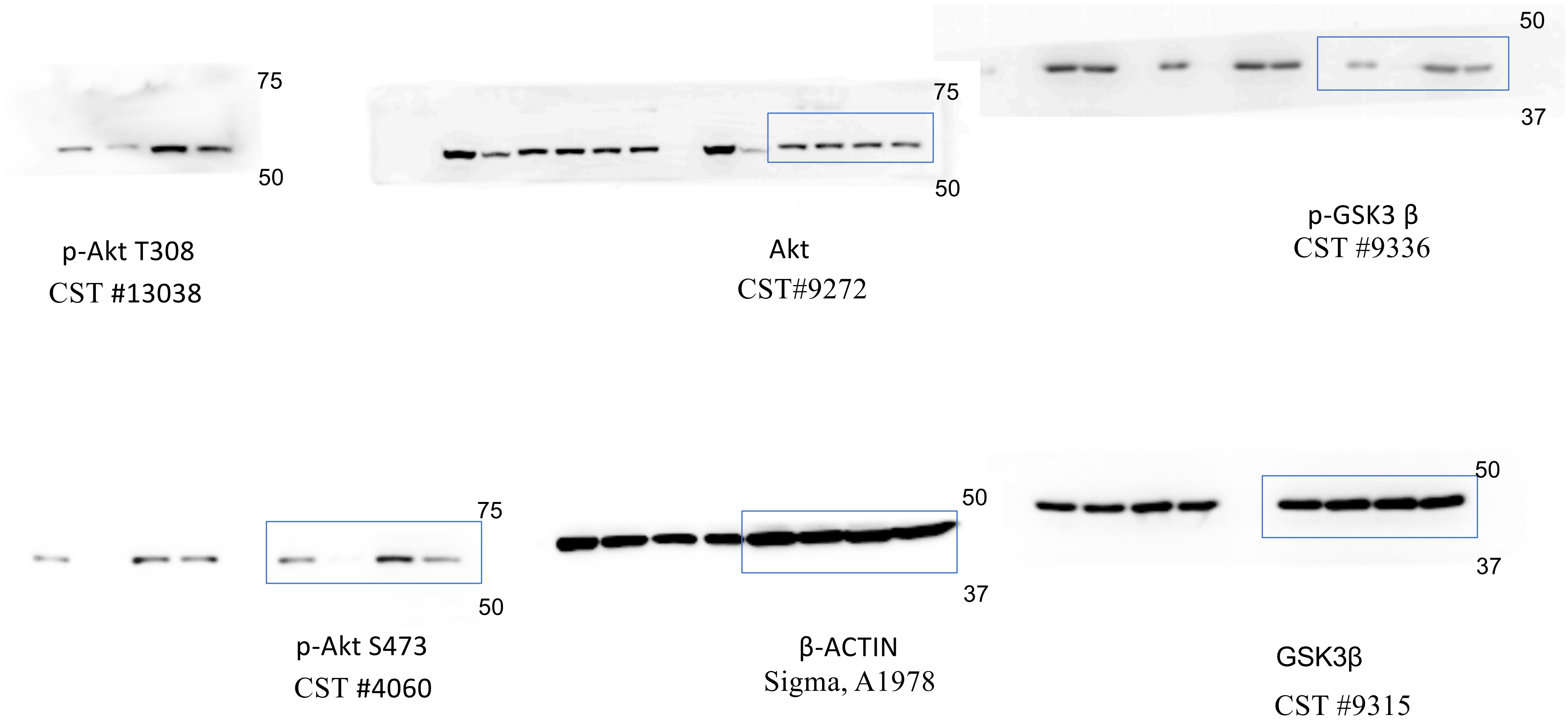

Supplement: Supplementary file 4 — Source Data [file 41467_2021_27921_MOESM4_ESM.zip › Uncut gels-reduced size.pdf]
